# RECIP 1.0 + PSA for response assessment in mCRPC patients treated with ^225^Ac / ^177^Lu PSMA combination therapy

**DOI:** 10.1186/s13550-025-01211-z

**Published:** 2025-03-04

**Authors:** Gabriel T. Sheikh, Astrid Delker, Mathias J. Zacherl, Adrien Holzgreve, Sarah L. Takayama Fouladgar, Marcus Unterrainer, Johannes Rübenthaler, Jozefina Casuscelli, Andrei Gafita, Lena M. Unterrainer

**Affiliations:** 1 Department of Nuclear Medicine, LMU University Hospital, LMU Munich, Munich, Germany; 2 Die Radiologie, Munich, Germany; 3 Department of Radiology, LMU University Hospital, LMU Munich, Munich, Germany; 4 Department of Urology, LMU University Hospital, LMU Munich, Munich, Germany; 5 Nuclear Medicine and Molecular Imaging Division, The Johns Hopkins Hospital, Johns Hopkins School of Medicine, Baltimore, USA; 6 Ahmanson Translational Theranostics Division, Department of Molecular and Medical Pharmacology, David Geffen School of Medicine, UCLA, Los Angeles, USA; 7 Bayerisches Zentrum für Krebsforschung (BZKF), partner site Munich, Munich, Germany

**Keywords:** Targeted alpha therapy, ^225^Ac, ^177^Lu, Radioligand therapy, RECIP, Tandem therapy

## Abstract

**Background:**

Targeted alpha therapy (TAT) with ^225^Ac has shown promising results in metastatic castration-resistant prostate cancer (mCRPC) patients pre-treated with [^177^Lu]Lu-PSMA radioligand therapy (RLT). A combination treatment regimen adding ^177^Lu to decreased ^225^Ac activities may improve toxicity profile while maintaining sufficient anti-tumor effect. We therefore evaluated clinical and image-based response parameters in patients treated with ^225^Ac-/^177^Lu-PSMA combination therapies (ALCT).

**Results:**

Complete response (RECIP-CR), partial response (RECIP-PR), stable disease (RECIP-SD), progressive disease (RECIP-PD) according to RECIP 1.0 was observed in 0/25 (0%), 12/25 (48%), 9/25 (36%) and 4/25 (16%) patients, respectively. Response by RECIP + PSA was observed in 14/25 (56%) patients and progression by RECIP + PSA in 8/25 (32%) patients. Interrater reliability for visual RECIP was substantial (κ = 0.757, *p* < 0.001), while agreement between visual and quantitative RECIP was almost fully congruent (κ = 0.879, *p* < 0.001). OS did not significantly vary among the four different therapy regimens (*p* > 0.05). When grouping patients with declining / stable PSA as responders, these patients showed no significant difference in overall survival compared to patients with progressive PSA after ALCT (*p* = 0.312). Similarly, there was no significant difference in median overall survival between patients without RECIP-progression (RECIP-PR + RECIP-SD) and patients with RECIP-progression (RECIP-PD) (*p* > 0.05), but when applying the composite classification, RECIP + PSA responders survived significantly longer compared to patients with RECIP + PSA progression (*p* = 0.049).

**Conclusions:**

ALCT is a promising therapeutic regimen that may prolong survival in patients who progress during [^177^Lu]Lu-PSMA RLT. Our results motivate to further investigate the use of RECIP + PSA as tool for response assessment and for overall survival prediction in mCRPC under ALCT.

**Supplementary Information:**

The online version contains supplementary material available at 10.1186/s13550-025-01211-z.

## Introduction

Prostate cancer is one of the most frequent malignancies in men worldwide [1]. In advanced prostate cancer specific binding and internalization of PSMA-targeting small molecules, allow for tumour-directed radiation therapy with beta- or alpha particle emitters. [^177^Lu]Lu-PSMA-617 and [^177^Lu]Lu-PSMA-I&T are the most common compounds used and RLT with [^177^Lu]Lu-PSMA-617 has recently been approved by the FDA as therapy for men with PSMA-positive metastatic castration-resistant prostate cancer (mCRPC), following confirmative efficacy results from the VISION trial [2]. Unfortunately, patients treated with beta emitting radio ligand therapy, relapse eventually [3, 4]. Targeted alpha therapy (TAT) with ^225^Ac has shown promising results even in this challenging group of recurrent prostate cancer patients, pre-treated with [^177^Lu]Lu-PSMA RLT [5, 6], but commonly induces xerostomia [6]. Kratochwil et al. found a treatment activity of 100 kBq per kilogram bodyweight to be a good compromise between efficacy and adverse events, amounting to 8 MBq for the average patient at normal nutrition [7]. Alternative regimens have been proposed to further improve tolerability without losing sufficient anti-tumor activity [8]. One promising approach to reach this goal is a combination treatment regimen adding ^177^Lu to decreased ^225^Ac activities [8–11]. Recently, a novel treatment response evaluation framework, factoring in PSMA-positive tumor volume and new lesions on PSMA-PET/CT (RECIP 1.0) as well as a composite classification, also taking into account biochemical response (RECIP + PSA), were developed and retrospectively validated in patients with mCRPC who had been treated with two cycles of [^177^Lu]Lu-PSMA RLT [12].

In our study, we aimed to assess the biochemical and imaging response of patients treated with combined ^225^Ac/^177^Lu PSMA RLT based on the abovementioned response evaluation framework.

## Materials and methods

### Patients

Patients treated with ALCT between March 2020 and May 2022 at the Department of Nuclear Medicine, LMU Munich were identified and included when meeting the following Inclusion criteria: advanced stage metastatic castration-resistant prostate cancer, failure of previous lines of treatment with contraindications to other approved treatment options or exhausted alternative treatment options, available baseline [^18^F]PSMA-1007-PET/CT as well as at least one follow up [^18^F]PSMA-1007-PET/CT after treatment with one or two homogeneous cycles of ALCT.

In accordance with the PROMISE V2 criteria PSMA-positivity of a lesion was defined as PSMA-expression above the uptake of the blood pool. For a patient to be deemed eligible for PSMA radioligand therapy all lesions above 1 cm in diameter had to show PSMA-expression above the spleen uptake.

Patients were selected for ALCT in case of progression under Lu-PSMA-RLT or after progression under chemotherapy in the case of diffuse marrow infiltration or visceral metastases (high tumor burden). Patients were assigned to one of three actinium-lutetium-combinations. Patients with high tumor burden received 8 MBq ^225^Ac and 1000 MBq ^177^Lu. The combination of 4/4000 MBq was given to patients with only nodal metastases and or disseminated bone metastases (low to intermediate tumor burden). 6/2000 MBq was administered in a minority of patients who would have been eligible for a combination of 8/1000 MBq but had the limitation of impaired kidney function or low bone marrow reserve. One patient received only 2/1000 MBq for tumor consolidation purposes in a very low tumor burden setting (1 ml TTV) and due to lack of supply.

RLT was performed based on § 13 (2b) an § 37 of the German Medicines Law and the updated Declaration of Helsinki concerning Unproven Interventions in Clinical Practice, respectively and patients were treated on a compassionate use basis after recommendation of our interdisciplinary urooncological tumor board. This retrospective study was approved by our institutional review board.

### PET/CT imaging

Baseline and follow-up [^18^F]PSMA-1007-PET/CT was performed on all patients as part of the clinical routine as previously described [13]. After giving written informed consent, an activity of 3 MBq [^18^F]PSMA-1007 per kilogram bodyweight was injected intravenously in accordance with the specific administration procedure [14] and the German Pharmaceutical Act § 13(2b). In case no contraindications were present, patients simultaneously received 20 mg of furosemide intravenously. Patients were scanned 60 min post tracer injection on a Biograph mCT PET/CT (Siemens Healthineers, Erlangen, Germany). A contrast enhanced CT scan in portal-venous phase with 1.5 ml Imeron 350 per kilogram bodyweight (Bracco Imaging, Milan, Italy) was followed by 2.5 min per bed position PET-acquisitions, which were iteratively reconstructed (TrueX, three iterations, 21 subsets) and smoothed with Gaussian post-reconstruction smoothing (2 mm full width at half maximum).

### ^225^Ac / ^177^Lu combination therapy

PSMA-I&T (Scintomics/ATT GmbH, Fürstenfeldbruck, Germany) was radiolabelled with ^225^Ac and ^177^Lu (both ITM Medical Isotopes GmbH, Munich, Germany) as described before [15, 16]. Since no prospective data on the ideal radionuclide combination was available combinations of 2 MBq ^225^Ac + 1000 MBq ^177^Lu (2/1000 MBq), 4 MBq ^225^Ac + 4000 MBq ^177^Lu (4/4000 MBq), 6 MBq ^225^Ac + 1000/2000 MBq ^177^Lu (6/1000 or 2000 MBq) or 8 MBq ^225^Ac + 1000 MBq ^177^Lu (8/1000 MBq) were administered to the individual patient based on clinical risk factors and tumor burden.

To reduce salivary gland perfusion, patients received cooling packs 30 min before to 4 h after radioligand injection. In addition, patients were given 50 mg p.o. prednisone per day for four days and ondansetron 4 mg p.o. + 2 l of isotonic saline solution on the day of therapy. Patients were hospitalized for at least 48 h post injection, in accordance with German radiation protection regulations. Patients with only one cycle, did not receive further cycles, either due to very good response or due to adverse events, precluding therapy continuation.

### Clinical and imaging parameters

Medical history was taken on prior androgen deprivation therapy (ADT), androgen receptor signalling inhibitors (ARSi), such as abiraterone and enzalutamide, taxane-based chemotherapy, Radium-223 therapy, poly(ADP-ribose)-polymerase inhibitor (PARPi) therapy and radiotherapy, the number of prior [^177^Lu]Lu-PSMA RLT cycles, the total of ALCT cycles during the period assessed, the activity administered per cycle and total activity administered.

Whole body (WB) PSMA-positive total tumor volume (TTV) and serum levels of prostate specific antigen (PSA [17]) were collected at baseline and follow up after 1–2 cycles of ALCT. New lesions, defined as any new focal uptake of PSMA ligand higher than the surrounding background, on follow up PSMA-PET/CT were documented. Overall survival (OS) was defined as time passed between the date of the first cycle of ALCT and the date of death or date of loss to follow-up.

### Response assessment

#### Imaging response

A novel response classification developed by Gafita et al. [12], “Response evaluation criteria in PSMA-PET/CT” (RECIP 1.0) was used for PET response assessment after 1–2 cycles of ALCT, taking into account PSMA-positive tumor volume (TTV), and whether or not new lesions can be found on follow-up PET/CT.

Based on the original publications, response was visually [18] and quantitatively [12] categorised into complete response (RECIP-CR, no residual PSMA-uptake, no new lesions), partial response (RECIP-PR, > 30% decrease of TTV, no new lesions), progressive disease (RECIP-PD, > 20% increase of TTV + new lesions) and stable disease (RECIP-SD, no response or progression as defined above or ≥ 30% decrease of TTV but new lesions or ≥ 20% increase of TTV but no new lesions). For a more relevant clinical distinction RECIP-CR/PR and RECIP-SD were merged into a non-progressive category (RECIP-nonPD).

Since the PET-Tracer and segmentation software (Affinity 3.0.1, Hermes Medical Solutions, Stockholm, Sweden) were different to the ones used in the original publication, a fixed threshold of 4.0 as cut-off for PSMA-positive tumor volume was applied for quantitative measurements, as previously described [19]. Off-target tissue was manually excluded. Visual assessment of tumor volume and the existence of newly developed metastases was carried out by two experienced readers, each with 5 years of experience reading PSMA-PET/CTs (GTS, LMU). For comparison with quantitative RECIP non-matching visual RECIP results were resolved by consensus.

#### PSA response

PSA response was assessed by comparing baseline PSA-values to values after 1–2 cycles of ALCT. In accordance with Gafita et al. [12] and the recommendations by the Prostate Cancer Clinical Trials Working Group 3 [20], a significant PSA-decline was defined as a decrease of ≥ 50% and significant progression as a PSA-increase of ≥ 25%. Values in between were considered stable. Composite imaging and biochemical response classification (RECIP + PSA) were also combined RECIP 1.0 with PSA-response to form a composite classification, as proposed by Gafita et al. [12]. Response was defined as RECIP-CR/PR or PSA-decrease ≥ 50% (RECIP + PSA-RD) and progression as RECIP-PD or PSA-increase ≥ 25% (RECIP + PSA-PD).

### Statistics

Statistics were calculated using IBM SPSS® Statistics (version 29; SPSS, Chicago, IL, USA). Statistical significance levels in case of a two-sided hypothesis were defined as *p* < 0.05, in case of a one-sided hypothesis as *p* < 0.025. Patient characteristics at baseline and follow up are presented as median (range). Normal distribution of data was evaluated with the Shapiro-Wilk test. Patient data at baseline and follow up time points were compared using the non-parametric, paired Wilcoxon test, while the Mann-Whitney-U-test was used to compare the different therapy groups. Spearman’s correlation coefficient was used to analyse correlations between different response parameters. To evaluate the agreement between visual and quantitative RECIP as well as the interreader reliability for visual RECIP Cohen’s Kappa was calculated. OS was plotted as Kaplan-Meier-plot and compared using the log-rank test.

## Results

### Patient characteristics

Twenty-five consecutive patients received a total of 65 cycles of ALCT (1–8 cycles/patient). The median number of cycles was two, four patients (16%) received one cycle, 21 patients (84%) received a minimum of two cycles. Patients were divided into four treatment groups as follows: Ten patients were treated with 8/1000 MBq, ten patients with 4/4000 MBq, four patients with 6/1000 or 2000 MBq and one patient with 2/1000 MBq for the first 1–2 cycles. Seven patients received ALCT immediately after failure of chemotherapy, while ALCT was applied only after failure of previous PSMA-directed therapy in 18 patients. The median activity applied was 5.97 (1.92–8.70 MBq) for ^225^Ac and 1073 (559–4328 MBq) for ^177^Lu. Eighteen patients received up to 10 cycles of standalone [^177^Lu]Lu-PSMA-RLT and two of those patients up to another 5 cycles of standalone [^225^Ac]-PSMA-TAT before receiving combination therapy. Other documented prior systemic therapies were androgen deprivation therapy in 20/25 (80%) patients, ARSi in 22/25 (88%) patients, docetaxel in 23/25 (92%) patients, cabazitaxel in 12/25 (48%) patients, ^223^Ra in 5/25 (20%) patients, and Olaparib in 1/25 (4%) patients. 15/25 (60%) patients underwent radical prostatectomy (RP) and 17/25 (68%) patients received either local or resalvage radiotherapy (RTx). At baseline, patients showed metastatic spread to the bone (25/25, 100%), lymph nodes (22/25, 88%), the liver (3/25, 12%), the lung (2/25, 8%) and the peritoneum (2/25, 8%). A baseline and follow-up [18 F]PSMA-1007-PET/CT was performed on all patients. The mean [18 F]PSMA-1007 activity applied was 242 MBq (range 154–276 MBq) for the baseline PET/CT and 223 MBq (170–310 MBq) for the follow-up PET/CT. The mean injection time was 61 min (range 50–93 min) and 68 min (range 51–85 min) for the baseline and follow-up PET/CT, respectively. Thirteen patients deceased during the observation period with a median OS of 11.86 months 95%CI [7.0-16.7]. For further specifications see Table 1.

**Table 1:** Patients characteristics

Number of patients treated		25
Median patient age		75 (60–87)
Number of ALCT-cycles	total	65
	per patient	1–8
	median	2
Median activity/cycle [MBq]	^225^Ac	5.97 (1,92 − 8,7)
	^177^Lu	1073 (559–4328)
Treatment groups [MBq ^225^Ac] / [^177^Lu]	2/1000	1/25
	4/4000	10/25
	6/1000 or 2000	4/25
	8/1000	10/25
Therapies prior to ALCT	RP	15/25
	RTx	17/25
	ADT	20/25
	ARSi	22/25
	docetacel	23/25
	cabazitaxel	12/25
	Ra-223	5/25
	PARPi	1/25
	[^177^Lu]Lu-PSMA RLT	8/25
	^225^Ac-PSMA TAT	2/25
Location of metastases	bone	25/25
	lymph nodes	22/25
	liver	3/25
	lung	2/25
	peritoneum	2/25

### PSA response

**Fig. 1:**
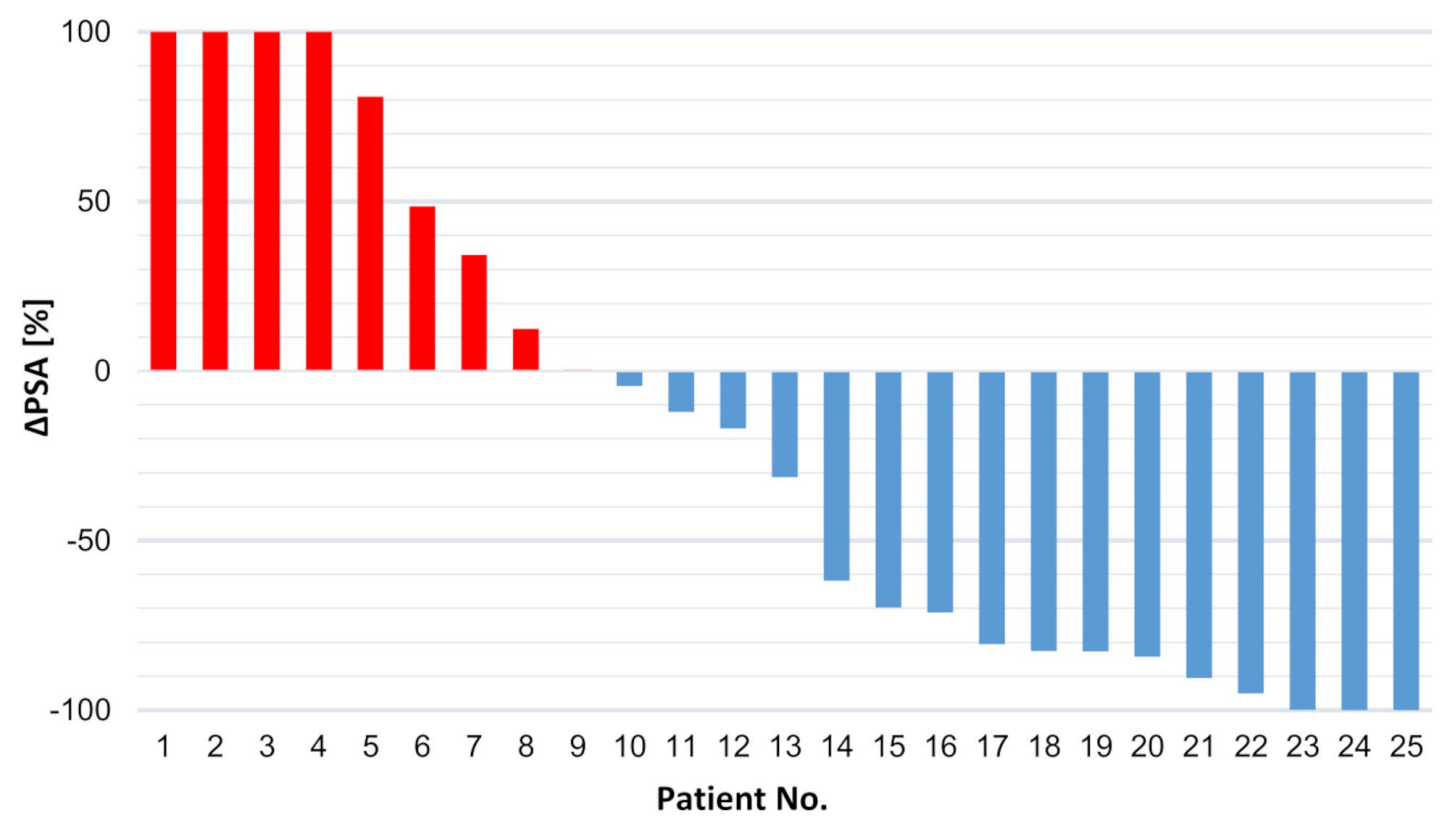
Waterfall plot of PSA response in percent after 1-2 cycles of ALCT

The median PSA-level was 132 ng/ml (range 0.03–1512 ng/ml) at baseline and 76.8 ng/ml (range 0-1330) at follow-up. 12/25 (48%) patients showed a PSA decrease of 50% or more (range − 100 to -62%) and 9/25 (36%) patients of 80% or more after one or two cycles of ALCT. 17/25 (68%) patients showed any PSA decline. 7/25 (28%) patients remained stable in respect to serum PSA level (range − 31 to + 12.5%) and 6/25 (24%) showed an increase of PSA-levels (range + 34% to + 743%) (Fig. 1). There was no significant difference between baseline and follow-up PSA levels (*p* = 0.313), between the different therapy groups (*p* = 0.592) or between patients with immediate ALCT and patients with ALCT after prior [^177^Lu]Lu-PSMA RLT and/or [^225^Ac]Ac-PSMA TAT (*p* = 0.198). We also did not find a significant difference in OS when comparing PSA non-progressive patients with either declining (≥ 50%) or stable PSA (11.9 mo, 95%CI [5.9–17.8]) to PSA progressive patients (5.3 mo), *p* = 0.316 (Supplement 1).

### Whole body PSMA-positive tumor volume

**Fig. 2:**
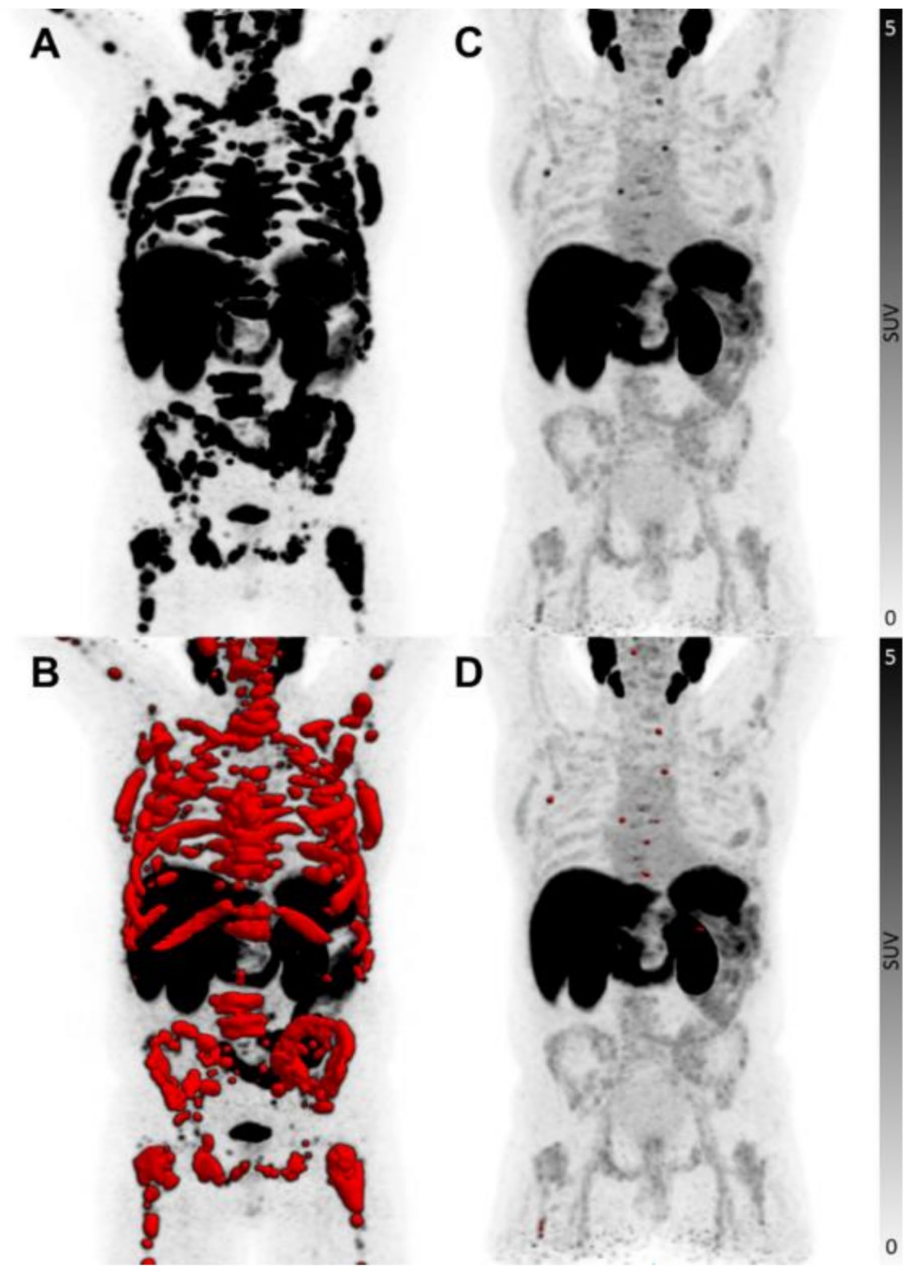
Example of a patient with TTV response. Maximum-intensity projections of the same patient before (**A**/**B**) and 3 months after ALCT (**C**/**D**). Over the same period, the PSA level decreased from 674 to 0.55 ng/ml. TTV is highlighted in red in images B and D

**Fig. 3:**
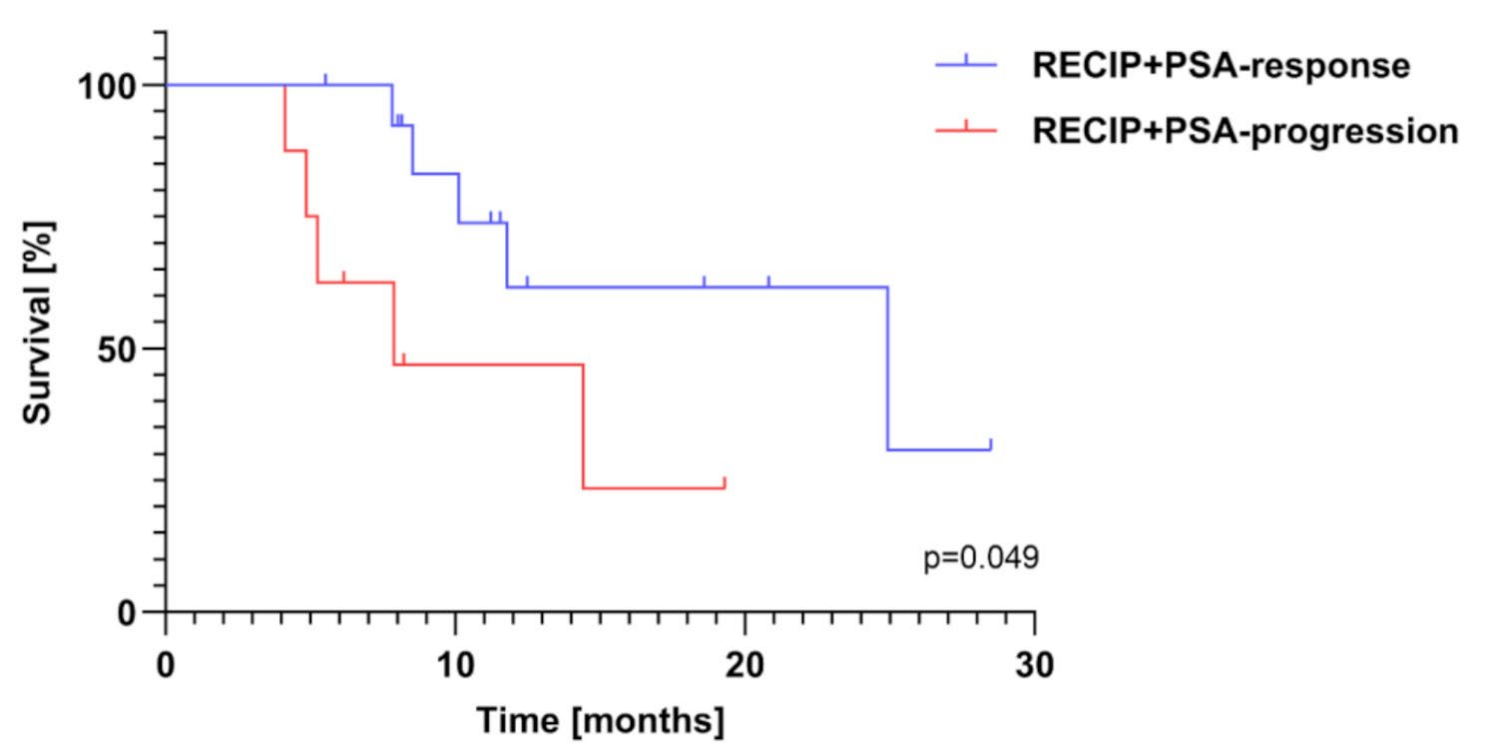
Kaplan-Meier plot showing overall survival of patients with tumor response (blue line) and tumor progression (red line) according to RECIP + PSA. The log rank test revealed a significant difference in overall survival between the two groups (*p* = 0.049)

The median TTV was 399 ml (range 0.91–2832 ml) at baseline and 244 ml (range 0.27–1370 ml) at follow up. 14/25 (56%) patients reached a minimum of 30% decrease of PSMA-positive tumour volume after a maximum of two cycles of ALCT (range − 100 to -46.5%) (Figs. 2), 3/25 (12%) patients were stable with tumour volumes between − 10.5% and + 10.5% and 8/25 (28%) patients had a significant increase in PSMA-positive tumour volume (range + 43% to + 398%) (Supplement 3). There was no significant difference between baseline and follow-up TTV (*p* = 0.093) or between the different therapy groups (*p* = 0.876), but patients with immediate ALCT had a significantly higher decrease of TTV compared to patients with ALCT after standalone [^177^Lu]Lu-PSMA RLT and/or [^225^Ac]Ac-PSMA TAT (*p* = 0.002).

### RECIP 1.0

RECIP-CR was observed in 0/25 (0%), RECIP-PR in 11/25 (44%), RECIP-SD in 8/25 (32%), and RECIP-PD in 6/25 (24%) patients. 12/25 (48%) patients had new lesions on follow up PSMA-PET/CT after 1–2 cycles of ALCT. Interrater reliability for visual RECIP 1.0 was substantial with a Kappa value of 0.757, *p* < 0.001 and agreement between visual and quantitative RECIP was almost fully congruent with a Kappa value of 0.879, *p* < 0.001. Patients with immediate ALCT were not significantly different to patients with ALCT after prior PSMA-directed therapy with regard to the RECIP categories applied (*p* = 0.074). There was no significant difference in OS between patients with RECIP-PR (24.9mo, 95%CI [9.9–39.9]), RECIP-SD (8.2mo, 95%CI [2.0-14.4]) or RECIP-PD (14.5mo, 95%CI [4.4–24.5]), *p* = 0.314 (Supplement 2). There was also no significant difference in OS when grouping together RECIP-PR and RECIP-SD patients as non-progressive disease (11.9mo, 95%CI [9.0-14.8]) and comparing them to patients with RECIP-PD, *p* = 0.851.

### RECIP + PSA composite classification

Applying the composite classification RECIP 1.0 + PSA, 14/25 (56%) patients showed response after 1–2 cycles of ALCT and 8/25 (32%) patients were progressive.

There was no significant difference in the RECIP + PSA composite classification of patients with immediate ALCT and patients with ALCT as last line after [^177^Lu]Lu-PSMA RLT and/or [^225^Ac]Ac-PSMA TAT (*p* = 0.141).

However, there was a significant difference in OS between RECIP + PSA responders (RECIP + PSA-RD, 24.9mo, 95%CI [5.7–44.1]) and patients with RECIP + PSA progression RECIP + PSA-PD (7.9mo, 95%CI [0.0-16.5]), *p* = 0.049 (Fig. 3).

## Discussion

We retrospectively evaluated the response of 25 patients with mCRPC treated with ALCT in respect to decline of PSA serum levels, the recently developed response classification RECIP 1.0, the composite classification RECIP + PSA, and OS.

ALCT, in which a reduced therapeutic ^225^Ac activity is compensated by the addition of ^177^Lu activity, has been proposed and partially confirmed to be a more tolerable, but equally effective alternative to PSMA TAT with ^225^Ac alone [8–11], even though the ideal ratio between the activities of both radionuclides still remains unclear.

RECIP 1.0 is a novel evidence-based response assessment framework recently developed by Gafita et al. that considers not only the dynamic change in tumor volume, defined as PSMA-positive tumor burden, but also the appearance of new lesions on follow-up PET/CT after [^177^Lu]Lu-PSMA RLT, combining both to group patients into four categories from complete response to progressive disease. Similarly to the consensus statement on PSMA-PET/CT response assessment criteria in prostate cancer [21] and against previous convention, the appearance of new lesions does not automatically lead to the categorization as progressive disease. RECIP 1.0 has been shown to have similar prognostic value to PSA response and the combination of both resulted in an even higher c-index, making the composite index RECIP 1.0 + PSA an even better biomarker for response to [^177^Lu]Lu-PSMA RLT [12]. RECIP 1.0 has been tested retrospectively only in mCRPC patients treated with [^177^Lu]Lu-PSMA RLT, but since [^225^Ac]Ac-PSMA TAT and ALCT share the same target and mechanism of action, there is a rationale to evaluate response using the same criteria. So far, RECIP 1.0 has shown several advantages in predicting response over other response assessment criteria in direct comparison [22], bone metastases for instance cannot reasonably be evaluated by RECIST or PERCIST.

It is important to keep in mind the advanced disease stage of our patient cohort as well as the mixed prior therapy regimens when interpreting the results even if – before receiving ALCT – all patients had been treated with standard guideline therapy (ADT, ARSi, and at least one taxane-based chemotherapy) and the majority of patients had already been treated with standalone [^177^Lu]Lu-PSMA RLT (72%) or [^225^Ac]Ac-PSMA TAT (8%).

PSA is an established biomarker for treatment response of mCRPC under systemic therapy. A recent study found early PSA response to PSMA-targeted radiotherapy to predict improved OS of mCRPC patients, irrespective of the extent of PSA decline [23]. The majority of our patients (68%) experienced any PSA decrease after one or two cycles of ALCT, 48% a minimum of 50% PSA decrease and 32% an even higher decrease of ≥ 80% PSA. Those results are in the region of those reported by different groups for ^225^Ac mono therapy (≥ 50% decrease in 14–63% [13, 15, 24]) and ALCT (43,5–65% [9, 11]), respectively and also match the values reported in the VISION trial (≥ 50% PSA decrease in 46% and ≥ 80% decrease in 33% [2]). While other studies evaluating predictors of prolonged OS after [^177^Lu]Lu-PSMA RLT found that PSA response had a significant impact on survival [25], there was no significant difference between PSA-responders and PSA-non responders in respect to OS after ALCT in our cohort.

TTV has been demonstrated to be an independent imaging biomarker in [^225^Ac]Ac-PSMA TAT [13]. A recently published consensus statement on PSMA PET/CT response assessment criteria in prostate cancer states that an increase of tumour volume > 30% should be considered progressive disease in polymetastatic prostate cancer, while an incomplete reduction of ≥ 30% should be called partial response [21]. The RECIP group uses an even lower threshold to define progressive disease (TTV ≥ 20%) after [^177^Lu]Lu-PSMA RLT, since they found it had the highest prognostic accuracy in respect to OS [26]. In our cohort 56% of patients showed a minimum of 30% decrease and 32% a minimum of 20% increase of PSMA-positive tumour volume after one or two cycles of ALCT. To our knowledge, TTV-response to ALCT has not been evaluated so far, but [^177^Lu]Lu-PSMA RLT based results from other groups found TTV partial response in 20–42% and TTV progression in 36–56% of patients [12, 27].

We used RECIP 1.0, as well as the composite framework RECIP + PSA to evaluate response in our patients after ALCT. 44% of our patients showed partial response and 24% progression after ALCT according to RECIP 1.0. More patients could be categorized as either responsive or progressive after ALCT using the composite framework (56% vs. 32%, respectively). Our RECIP-based response rates for ACLT therefore are similar or even higher than those previously reported for [^177^Lu]Lu-PSMA RLT by Gafita et al. and Kind et al. [27] ranging between 16 and 31% partial response and 31–56% progressive disease in their cohorts.

The median OS of our patients after ALCT was 11.9 months, which is well in line with the 11.1 months reported by Khreish et al. [11]. Even though patients with RECIP-PR had a longer median OS of 24.9 months compared to 14.5 months for RECIP-PD, there was no significant difference in OS between the RECIP categories; this might be the case due to the limited sample size in the evaluated patients. We also attribute the somewhat odd results that patients with RECIP-SD demonstrated a lower median OS of 8.2 months, compared to patients with RECIP-PD with a median OS of 14.5 months, to statistical effects due to the overall low number of patients. From a clinical perspective, only progressive disease requires a change of treatment strategy. We therefore also evaluated OS of non-responders (RECIP-PD) compared to therapy responders (RECIP-PR + -SD), but did not find any significant difference between those two groups either (*p* = 0.36). This contrasts with data reported by Gafita et al. and Kind et al. who reported significant differences in OS between responders and non-responders among their patients treated with [^177^Lu]Lu-PSMA RLT. However, there was significantly longer OS of responsive patients (PR + SD) compared to patients with progressive disease using the composite classification system RECIP + PSA (*p* = 0.049), similar to results reported by Gafita et al. and Kind et al. for [^177^Lu]Lu-PSMA RLT.

It is interesting to note that immediate ALCT following the failure of chemotherapy appears to exert a considerably more pronounced effect on TTV than ALCT subsequent to prior PSMA-RLT and/or PSMA-TAT. Identifying the underlying mechanism behind this phenomenon could help further improve treatment outcome through optimized treatment sequencing. However, it does not appear to have a significant impact on RECIP classification or overall survival, which may be due to the small size of our cohort.To summarize, ALCT showed a response pattern roughly similar to other PSMA-targeted therapies, but neither PSA-response nor RECIP 1.0 alone could prognosticate survival in our cohort, while the composite response classification RECIP + PSA indicated a longer OS after ALCT. These results highlight the potential impact of incorporating both PSMA-PET/CT by RECIP 1.0 and serum PSA levels in evaluating efficacy of PSMA-targeted RLT in daily practice.

The main limitations of our study are the retrospective design and the small patient cohort, especially the low number of patients deceased during the observation period, a significant limitation regarding the statistical power of our data. The heterogeneous therapy regimen as well as different prior therapies in this pilot patient cohort represents another limitation, even though we did not find any significant difference between the groups regarding response.

## Conclusion

ALCT might be an effective treatment in mCRPC patients pretreated with multiple systemic therapies and who progressed after standalone beta emitting RLT. The RECIP 1.0 and RECIP + PSA systems, combining different independent prognostic markers (PSA-response, total tumour volume, new lesions) could be a comprehensive tool useful for response assessment and risk stratification in patients who underwent ALCT. Our findings in this pilot cohort need to be further substantiated with additional prospective, clinical data from larger patient cohorts.

## Electronic supplementary material

Below is the link to the electronic supplementary material.

Supplementary Material 1

## Data Availability

Research data are stored in an institutional repository and will be shared upon request to the corresponding author.

## Abbreviations

ADT: Androgen deprivation therapy
ALCT: ^225^Ac-/^177^Lu-PSMA combination therapy
ARSi: Androgen-receptor signal inhibitor
CR: Complete response
CT: Computed tomography
MBq: Megabecqerel
mCRPC: Metastatic castration-resistant prostate cancer
OS: Overall survival
PARPi: Poly-ADP ribose polymerase inhibitor
PD: Progressive disease
PET: Positron emission tomography
PR: Partial response
PSA: Prostate-specific antigen
PSMA: Prostate-specific membrane antigen
RECIP: Response evaluation criteria in PSMA-PET/CT
RLT: Radioligand therapy
RTx: Radiation therapy
SD: Stable disease
TAT: Targeted alpha therapy
TTV: Total tumor volume
WB: Whole body
