# Supplementary material for: RECIP 1.0 + PSA for response assessment in mCRPC patients treated with 225Ac / 177Lu PSMA combination therapy
Source: EJNMMI Res. 2025 Mar 4;15:19. doi: 10.1186/s13550-025-01211-z (PMC11880445; doi:10.1186/s13550-025-01211-z)
Supplement: Supplementary file 1 — Supplementary Material 1 [file 13550_2025_1211_MOESM1_ESM.docx]

**SUPPLEMENTALS:**

**Supplement 1:** Kaplan-Meier plot showing overall survival of patients with PSA non progression (blue line) and PSA progression (red line) after ALCT. There was no significant difference in overall survival between the two groups (p=0.316).


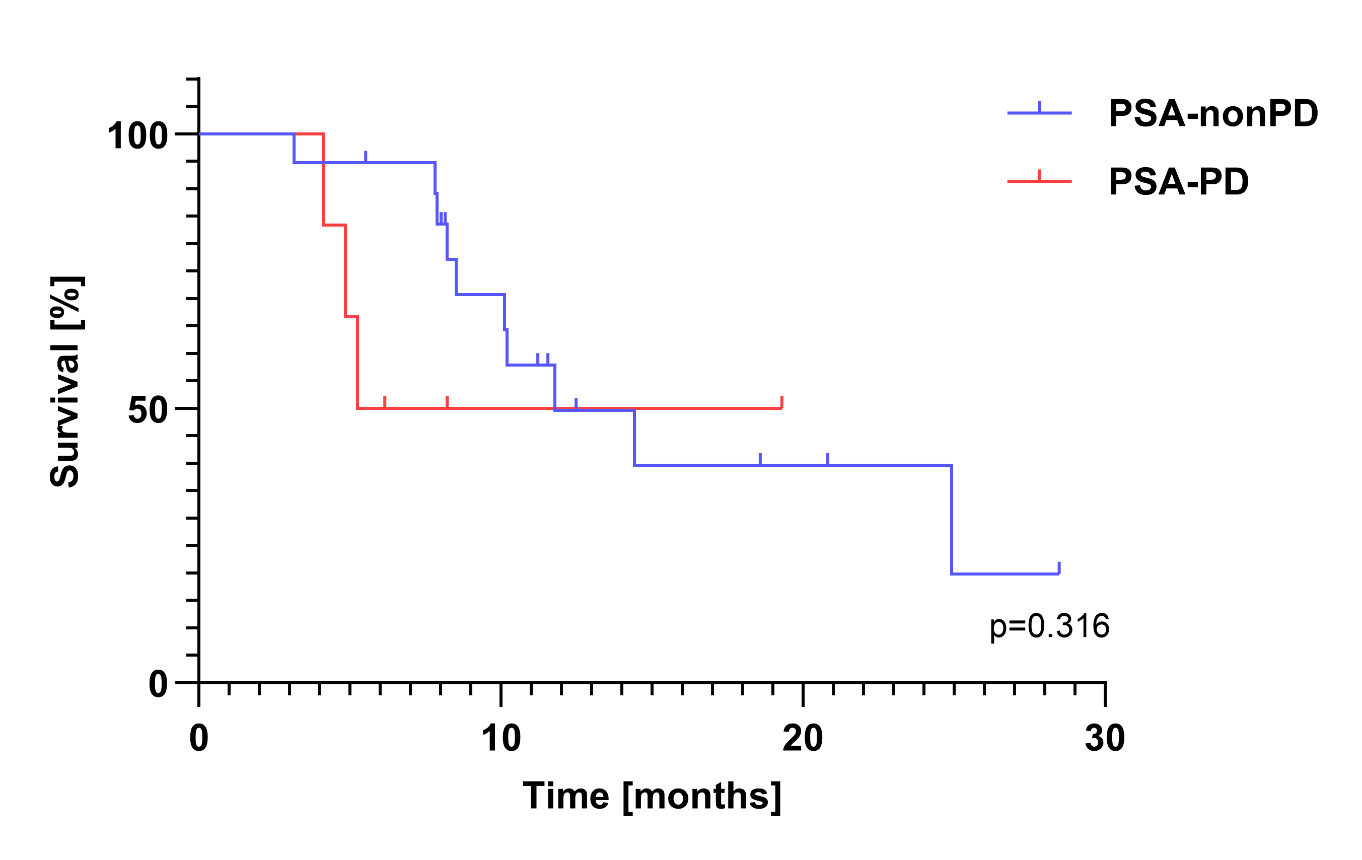


**Supplement 2:** Kaplan-Meier plot showing overall survival of patients with RECIP 1.0 partial response (blue line), stable disease (green line) progressive disease (red line) after ALCT. There was no significant difference in overall survival between the three groups (p=0.314).
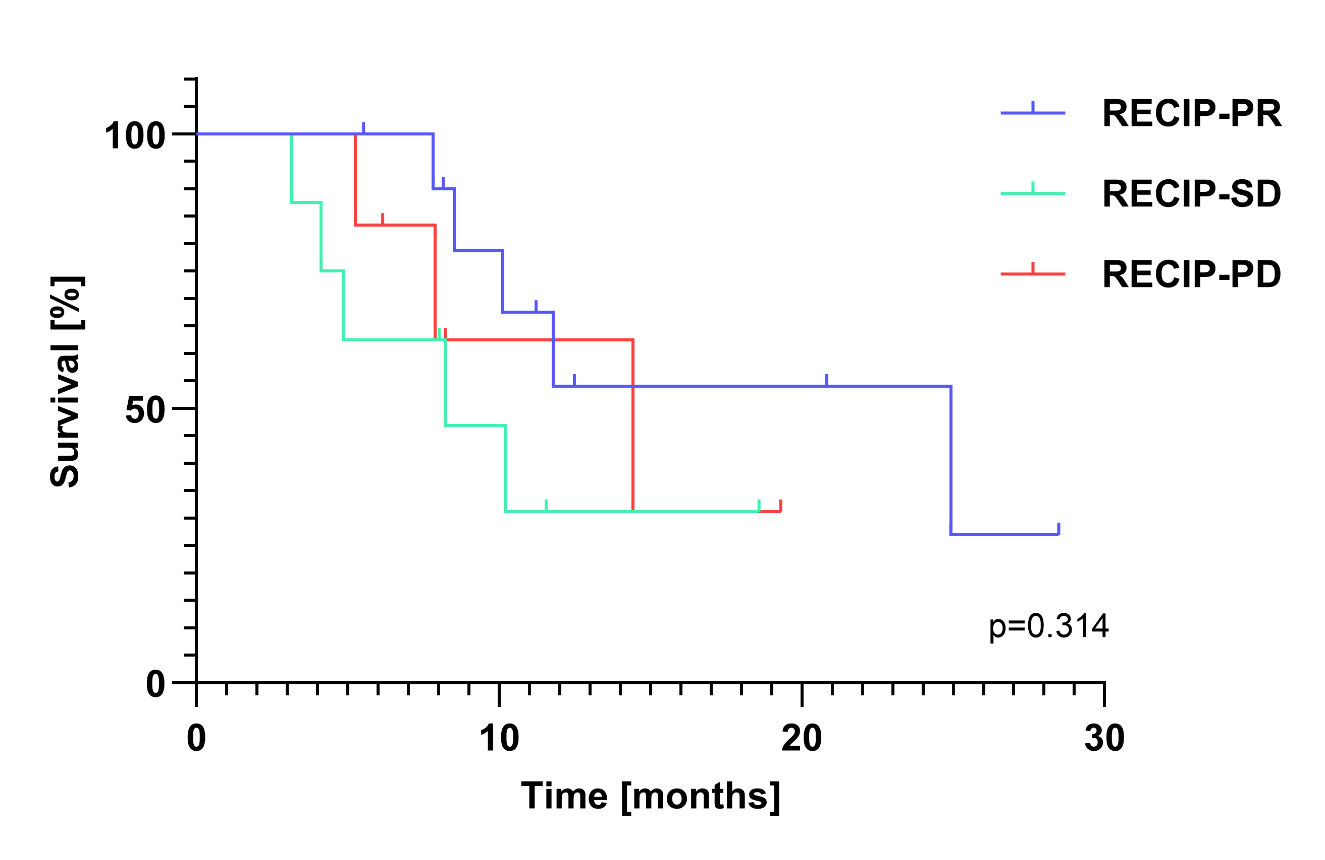


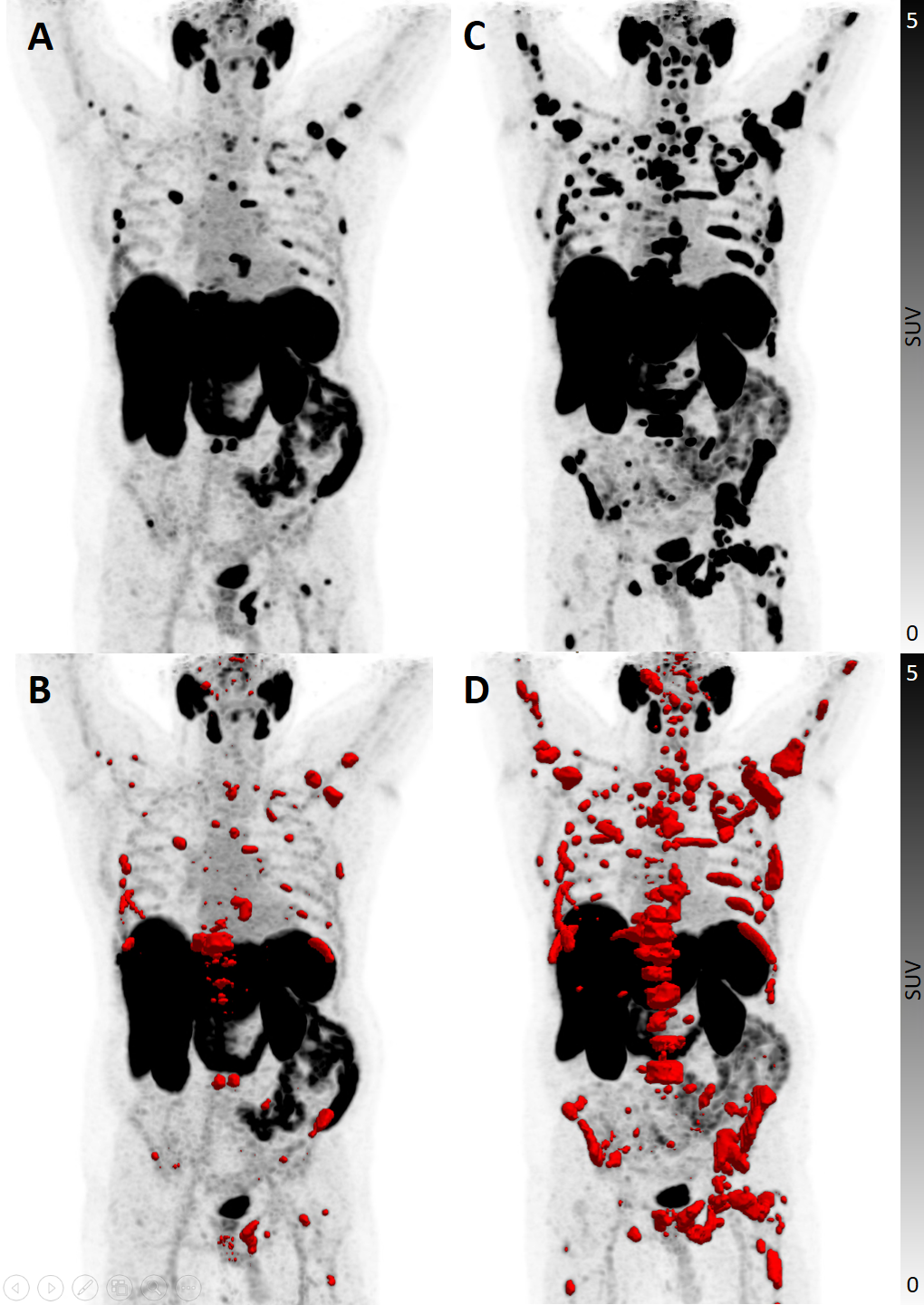
**Supplement 3**: Example of a patient with TTV progression. Maximum-intensity projections of the same patient before (A/B) and 3 months after ALCT (C/D). Over the same period, the PSA level increased from 15.3 to 129 ng/ml. TTV is highlighted in red in images B and D.
